# Perceptual training yields rapid improvements in visually impaired youth

**DOI:** 10.1038/srep37431

**Published:** 2016-11-30

**Authors:** Jeffrey B. Nyquist, Joseph S. Lappin, Ruyuan Zhang, Duje Tadin

**Affiliations:** 1 Vanderbilt Vision Research Center and Department of Psychology, Vanderbilt University, Nashville TN, 37240, USA; 2 Department of Psychology, Northern Michigan University, Marquette, MI, 49855, USA; 3 Neurotrainer, Marquette, MI, 49855, USA; 4 Center for Magnetic Resonance Research, Department of Radiology, University of Minnesota at Twin Cities, Minneapolis MN, 55455, USA; 5 Department of Brain & Cognitive Sciences and Center for Visual Science, University of Rochester, Rochester, NY, 14627, USA; 6 Department of Ophthalmology, University of Rochester School of Medicine, Rochester, NY, 14642, USA

## Abstract

Visual function demands coordinated responses to information over a wide field of view, involving both central and peripheral vision. Visually impaired individuals often seem to underutilize peripheral vision, even in absence of obvious peripheral deficits. Motivated by perceptual training studies with typically sighted adults, we examined the effectiveness of perceptual training in improving peripheral perception of visually impaired youth. Here, we evaluated the effectiveness of three training regimens: (1) an action video game, (2) a psychophysical task that combined attentional tracking with a spatially and temporally unpredictable motion discrimination task, and (3) a control video game. Training with both the action video game and modified attentional tracking yielded improvements in visual performance. Training effects were generally larger in the far periphery and appear to be stable 12 months after training. These results indicate that peripheral perception might be under-utilized by visually impaired youth and that this underutilization can be improved with only ~8 hours of perceptual training. Moreover, the similarity of improvements following attentional tracking and action video-game training suggest that well-documented effects of action video-game training might be due to the sustained deployment of attention to multiple dynamic targets while concurrently requiring rapid attending and perception of unpredictable events.

Perceptual training can lead to considerable improvements in our sensory, attentional and cognitive abilities^1^,^2^. Arguably, the potential utility of perceptual training is highest in individuals who have diminished perceptual abilities. Individuals with low vision are of particular interest. In the United States, more than 20 million, including over 500,000 children, have difficulty seeing even when wearing glasses or contact lenses^3^. Many visual impaired children require special education services^4^. A host of specific etiologies are responsible for disabling vision, including amblyopia, retinopathy of prematurity, congenital nystagmus, ocular albinism, etc. These conditions are often referred to as low vision (LV). Unfortunately, in traditional clinical practice, it is believed that few visual impairments respond to therapeutic techniques. Instead of adjusting the visual deficit itself, the focus is typically on adjusting the individual to the world through instruction of everyday skills. Perceptual learning studies, however, suggest a reconsideration of these traditional views. For typically-sighted individuals, perceptual learning can improve a wide range of visual abilities, including perception of motion, orientation, spatial frequency, as well as vernier acuity, identification of objects, visual search and face perception^1^,^2^. Visually impaired youth and adults also show evidence of perceptual learning, including improved contrast sensitivity, acuity thresholds, feature search and reading speeds^5^,^6^,^7^.

Early work on perceptual learning has found strong evidence for highly focused visual improvements that are specific to the trained parameters^1^,^2^,^8^. However, recent studies revealed a number of cases where perceptual learning can transfer to untrained tasks and stimuli^9^,^10^. In fact, a significant portion of the current research on perceptual learning is aimed toward elucidating conditions that facilitate generalization of perceptual training. Effects of perceptual learning also vary across people. Notably, for low vision individuals, emerging evidence indicates that perceptual training can yield broader transfer to untrained stimuli and tasks^11^,^12^,^13^. Low vision individuals are also more likely to have sizeable benefits from perceptual training, with learning effects tending to be larger for those with poor initial performance^14^,^15^,^16^. In sum, there is good evidence that perceptual training can be an effective intervention for low vision.

A related line of research shows that video game training can result in widespread improvements^1^ that include both higher-level attention tasks^17^,^18^,^19^,^20^ as well as low-level visual tasks, such as contrast sensitivity^21^ and orientation discrimination^22^. Recent work has demonstrated the therapeutic effectiveness of video game training in visually impaired individuals^23^,^24^,^25^,^26^,^27^. Action video games, unlike traditional perceptual learning tasks, are much more complex in their task demands and stimuli. These games require rapid deployment of attention to multiple, and often unpredictable, dynamic stimuli. This, in turn, places additional demands on dynamic control of visuomotor mechanisms, which may be particularly important in peripheral vision^28^,^29^. LV is often associated with a characteristic reduction in attention to peripheral visual fields, even when there is no known impairment to this region^30^,^31^,^32^; impairments of either the central or peripheral fields often reduce visual performance in using other regions^33^,^34^. This suggests that some of the impairments observed in LV might reflect higher-level attentional deficits—deficits that might be particularly amendable via action video game training.

Here, we investigated the effects of perceptual training on visual functioning of children with low vision by contrasting three different training regimens: a typical action video game (AVG), a novel modified attentional tracking task (MAT) and a control, non-action video game. The tracking task combined conventional attentional tracking^35^,^36^ with a spatially and temporally unpredictable motion discrimination task. It was designed to emulate a specific subset of task demands found in action video games. In general, an effective video-game player must be able to distribute and switch his/her attention to many locations in rapid succession while at the same time monitor the display for unexpected targets. Targets also move, requiring players to track multiple objects simultaneously, while ignoring irrelevant stimuli. The attentional tracking training task was designed to present these specific task characteristics in a controlled fashion, while eliminating many other components of action video games, such as the demand for speeded hand-eye coordination, complex stimulus parameters, and large variations in task demands.

By comparing these three training regimens (AVG, MAT and control), we can directly test whether the laboratory-style attentional tracking task includes sufficient visual demands to produce training effects similar to those found in action video games. As we are interested in training paradigms that work relatively quickly, the training was limited to ~8 hours (10 sessions). Training effects were evaluated by several psychophysical measures of visual functioning in both central and peripheral visual fields. Motion perception at various visual field locations was assessed with a direction discrimination task. The spatial resolution of attention was assessed using a perceptual crowding task^37^. A visual search task of naturalistic scenes was included to assess more ecological forms of visual functioning. Finally, for a subset of participants, we also examined whether training-induced improvements remained stable over time by conducting follow-up testing 12 months after the end of training.

## Results

### Pre-training results

We reported a detailed analysis of pre-training results in a previous paper^32^. In brief, pre-training results revealed low central acuity for motion, deficits in motion perception that increased with increasing visual eccentricity, large impairments in the visual search ability and moderate deficits in visual crowding. These impairments exhibited only a modest relationship with visual acuity. Overall, the results were consistent with abnormal attentional mechanisms playing a significant role in observed task impairments^32^.

**Figure 1:**
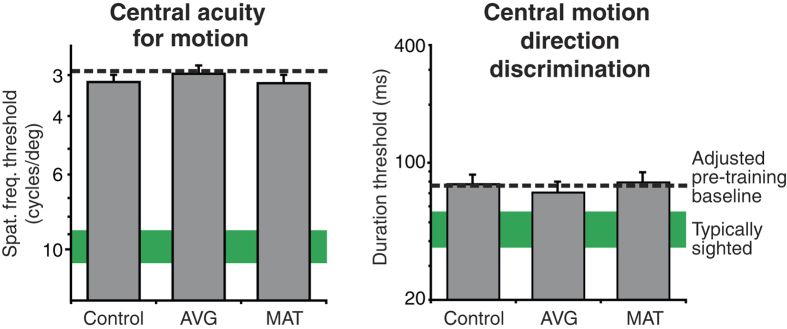
Effects of training on foveal motion perception. Estimated post-training thresholds for three training groups in the central acuity for motion task (left) and central motion discrimination task (right). To be consistent with other graphs in this paper, Y-axis for the acuity task is flipped (i.e., larger numbers, indicating better performance, are given at the bottom of the y-axis). Error bars are SEM. Adjusted pre-training baseline for LV participants is shown with a dashed line. Mean performance for TS participants is shown with green horizontal bars. The width of the bar includes mean +/− SEM.

**Figure 2:**
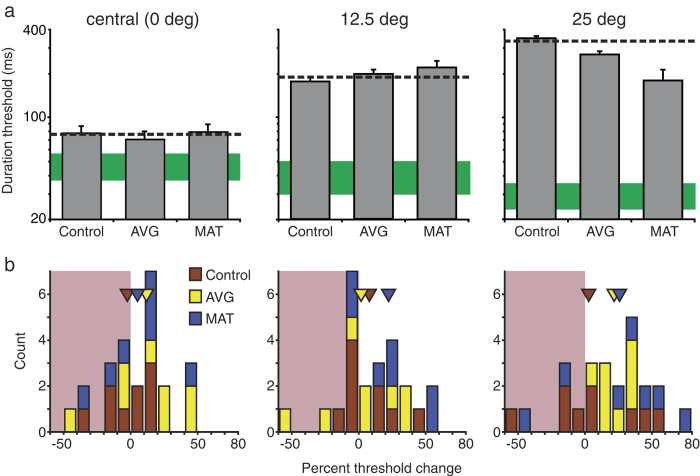
Effects of training on single target motion direction discrimination. (**a**) Estimated post-training thresholds for three training groups (grey bars). For comparison purposes, central motion discrimination results from Fig. 1 are replotted (left panel). All conventions are as described in the Fig. 1 legend. Note that while performance for TS individuals improved with eccentricity, performance of LV individuals worsens^32^. (**b**) Distribution of threshold changes from pre to post-training for individual participants from three training groups. Positive numbers indicate improvements. Triangles show mean results for three training groups. The leftmost bin includes data between −85 and −50. Shaded reddish areas indicate decreases in performance.

**Figure 3:**
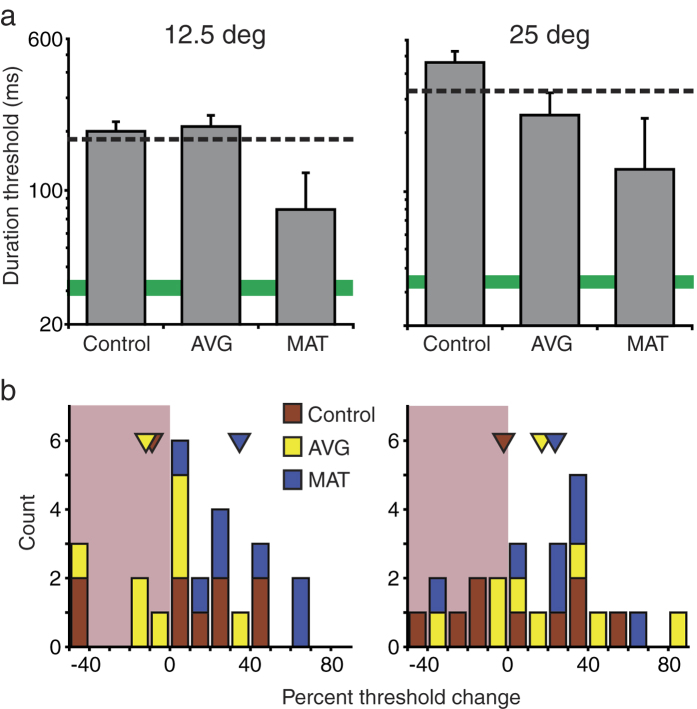
Effects of training on multi-target direction comparison. (**a**) Estimated post-training thresholds for three training groups (grey bars). All conventions are as described in the Fig. 1 legend. (**b**) Distribution of threshold changes from pre to post-training for individual participants from three training groups. Positive numbers indicate improvements. Triangles show mean results for three training groups. The leftmost bin includes data between −180 and −40.

**Figure 4:**
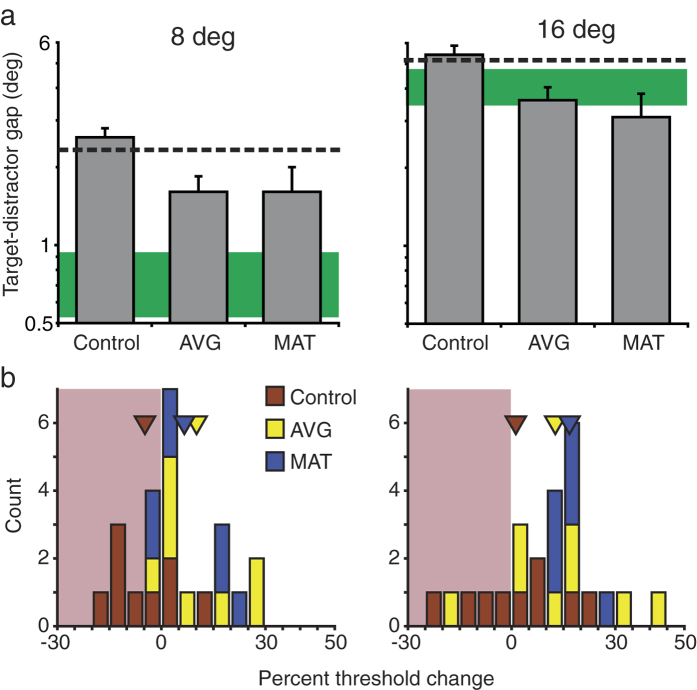
Effects of training on visual crowding. (**a**) Estimated post-training thresholds for three training groups (grey bars). All conventions are as described in the Fig. 1 legend. (**b**) Distribution of threshold changes from pre to post-training for individual participants from three training groups. Positive numbers indicate improvements. Triangles show mean results for three training groups.

**Figure 5:**
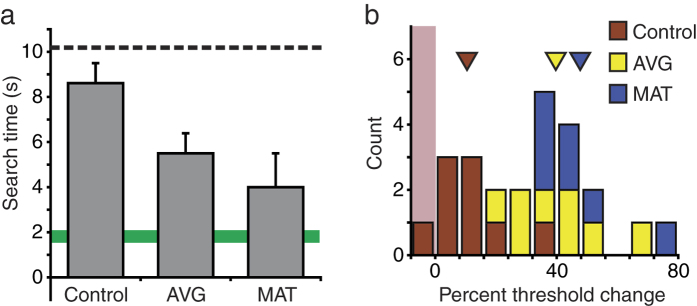
Effects of training on visual search. (**a**) Estimated post-training thresholds for three training groups (grey bars). All conventions are as described in Fig. 1 legend. (**b**) Distribution of threshold changes from pre to post-training for individual participants from three training groups. Positive numbers indicate improvements. Triangles show mean results for three training groups.

These results constitute a baseline against which training effects were evaluated. In Figs 1, 2, 3, 4, and 5, pre training data are indicated by dashed lines (for LV; n = 24) and green solid bars (for typically sighted, TS, n = 7). To deal with a considerable amount of baseline variability among LV participants, pre-training task performance was included as a covariate for each analysis.

### Performance on training tasks

After ten training sessions, all participants improved on their respective training tasks: AVG (n = 7), MAT (n = 8) and a control game (n = 9) that is similar to the popular game of Tetris (see Methods for detailed training task descriptions). Because game performance is unlikely to fall on a ratio scale, improvement numbers listed below are for illustration only. Moreover, because changes in task difficulty were not matched for the three training tasks and for different MAT measures, performance among training tasks cannot be directly compared (e.g., it is much easier to gain a 10% improvement on the control task than on the MAT task). Improvement for the AVG group was tracked by the descriptive statistics for the game. On average, participants completed 45% of “challenges,” with 2.4 “tournaments” per training session. Improvement for the MAT group was tracked by four different measures, three for the tracking task (ball velocity, number of tracked targets, and number of distractors) and one for the unpredictable motion discrimination task (stimulus duration). To be conservative, we used the second training session as the baseline. Participants exhibited large improvements from the first to the second session, but we suspect that, at least in part, these improvements also reflected learning of task demands. Comparing the second and the final training session, these four measures improved by 24%, 12%, 12% and 13%, respectively. Performance in the control video game was measured for each session by the median score of all games played in that session and revealed a 376% improvement over 10 training sessions.

### Post-training results

The effects of training were assessed by ANCOVA and MANCOVA analyses. In all analyses reported below, age, acuity and pre-training thresholds were included as covariates. All pairwise comparisons are Bonferroni corrected.

### Foveal acuity for motion and foveal motion direction discrimination

Different training regimens had no significant effect on the visual performance in either of these foveal tasks (Fig. 1). This was expected because both experimental training tasks were designed to place high demands on peripheral visual processing. Foveal spatial acuity was analysed with a 3 × 2 ANCOVA, including training group (control, AVG or MAT) and nystagmus (nystagmus or no nystagmus) as between-subjects factors. There was no significant effect of training group (F_2,15_ = 0.94, p = 0.41, observed power = 0.18). The nystagmus factor and training group by nystagmus interaction were also not significant (F_2,15_ = 0.41, p = 0.53, observed power = 0.09; F_2,15_ = 1.10, p = 0.36, observed power = 0.21, respectively). An equivalent analysis for the foveal motion direction discrimination performance also showed that different training regimens had no significant effect on the visual performance in this task (F_2,15_ = 2.83, p = 0.09, observed power = 0.47). Here, the nystagmus factor was significant (F_2,15_ = 5.85, p = 0.029, observed power = 0.62)—participants with nystagmus exhibited positive training-induced changes relative to those without nystagmus (on average, a 11% improvement vs. 4% worsening, respectively). The group by nystagmus interaction was not significant (F_2,15_ = 1.84, p = 0.92, observed power = 0.32).

### Single target motion direction discrimination

Potential effects of perceptual training on peripheral vision were a main focus of this study. Results (Fig. 2) show that both AVG and MAT resulted in improved motion discrimination performance at 25° but not at 12° eccentricity (Fig. 2). Data were analysed with a 3 × 2 × 2 MANCOVA, with training group (control, AVG, MAT) and nystagmus as a between-subjects factor and eccentricity (12° and 25°) as a within-subjects factor. The main effect of training group was statistically reliable (F_2,14_ = 4.14, p = 0.039; observed power = 0.63). Pairwise comparisons revealed significantly better performance in the MAT group relative to the control group (p = 0.045, Bonferroni corrected), with no significant differences between AVG and control groups (p = 0.30) and between MAT and AVG groups (p = 0.43). The main effect of nystagmus was not significant (F_1,14_ = 0.33, p = 0.58, observed power = 0.08). Also, we noted a strong interaction of training and eccentricity (F_2,19_ = 9.34, p = 0.004; observed power = 0.94). Pairwise comparisons revealed no significant differences between control and experimental groups at near periphery (AVG, p = 0.82; MAT, p = 0.47) but significant differences at far periphery (AVG, p = 0.032; MAT, p = 0.002, Bonferroni corrected).

### Multi-target direction comparison

Training also improved participants’ ability to compare motion directions of spatially separate stimuli (Fig. 3). Data were analysed with a 3 × 2 × 2 MANCOVA, with training (control, AVG, or MAT) and nystagmus as between-subjects factors and eccentricity (12° vs. 25°) as a within-subjects factor. There was a marginal effect of training group, F_2,14_ = 3.69, p = 0.052; observed power = 0.58). Nystagmus was not a significant factor (F_1,14_ = 0.013, p = 0.91, observed power = 0.05). Notably, there was a significant interaction between training group and eccentricity (F_2,14_ = 4.27, p = 0.036; observed power = 0.65), with experimental training groups separating from the control group in the far periphery (Fig. 3). Pairwise comparisons revealed no significant differences between control and experimental groups at near periphery (AVG, p = 1.0; MAT, p = 0.13). At far periphery, there was no significant difference between control and AVG groups (p = 0.19) and a marginal difference between control and MAT groups (p = 0.07, Bonferroni corrected).

### Visual crowding

AVG and MAT training also improved participants’ tolerance for spatial crowding (Fig. 4). Data were analysed with a 3 × 2 × 2 repeated measures MANCOVA with training (control, action, or psychophysical) and etiology (nystagmus vs. not nystagmus) as between-subjects factors and eccentricity (8° and 16°) as a within-subjects factor. There was a main effect of training group (F_2,14_ = 5.77, p = 0.015; observed power = 0.782). Pairwise comparisons revealed that the control group was significantly worse than the AVG group (p = 0.025) and marginally worse than the MAT group (p = 0.064, Bonferroni corrected), with no significant differences between AVG and MAT groups (p = 1.0). Nystagmus was not a significant factor (F_1,14_ = 0.39, p = 0.54, observed power = 0.09). A marginal interaction between training and eccentricity was noted (F_2,14_ = 3.29, p = 0.067, observed power = 0.53). At the near location (8°), post-training performance for AVG group was significantly better than that of the control group (p = 0.042, Bonferroni corrected), with no significant differences between MAT and control groups (p = 0.19). Relative to the control group at the far location (16°), we observed a significant improvement for the AVG group and a marginal improvement for the MAT group (p = 0.034, 0.059, respectively, all Bonferroni corrected).

### Visual search

Large training-induced improvements were observed for the visual search task (Fig. 5). Visual search performance was analysed with a 3 × 2 ANCOVA, including training group (control, AVG or MAT) and nystagmus as between-subjects factors. There was a significant effect of training group (F_2,15_ = 4.49, p = 0.03; observed power = 0.68). Here, there were marginal differences between both AVG and MAT groups and the control group (p = 0.081, 0.067, respectively, Bonferroni corrected), with no differences between AVG and MAT groups (p = 1.0). The nystagmus factor was not significant (F_1,15_ = 0.63, p = 0.44; observed power = 0.12), with no significant training by nystagmus interaction (F_2,15_ = 1.35, p = 0.29; observed power = 0.25).

### Summary of results

**Figure 6:**
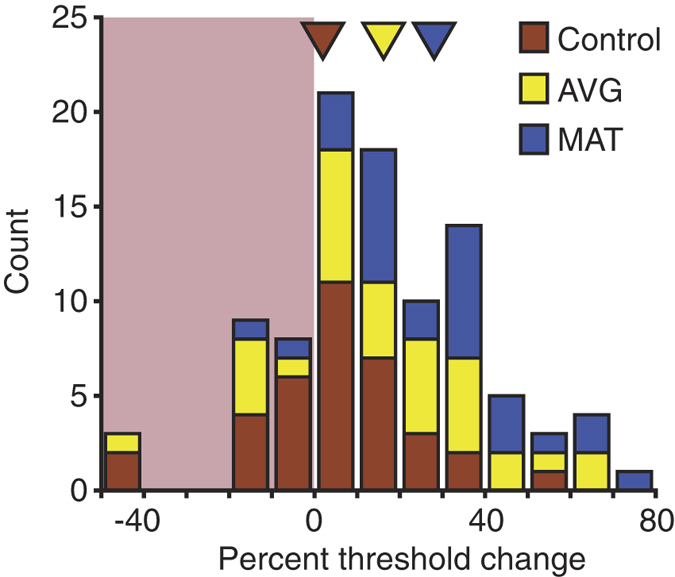
Pooled over tasks involving visual periphery, distribution of training-induced threshold changes for individual participants from three training groups. For tasks with two stimulus eccentricities (visual crowding, single target motion direction discriminations and multi-target direction comparison), we used the average over the two eccentricities. For visual search, we used the data shown in Fig. 5b. Positive numbers indicate improvements. Triangles show mean results for three training groups. The leftmost bin includes data between −150 and −40.

For all tasks where task demands involve attention to peripherally presented stimuli, we found significant effects of training. This was evident in significant main effects of training group and/or significant training group by eccentricity interactions. Figure 6 summarizes these results by showing distribution of training-induced threshold changes for individual participants pooled over tasks involving visual periphery. While distribution for the control tasks centres on 0 (2.1% threshold improvement), AVG and MAT task results are biased toward threshold improvements (16.4% and 28.4% average threshold improvements, respectively).

Motivated by evidence that larger perceptual training effects are often observed for individuals with poor pre-training performance^14^,^15^,^16^, we examined correlations between pre-training performance and training-induced improvements. Here, we combined AVG and MAT groups to increase statistical power. The results revealed no significant correlations over all tasks and conditions, with just one marginal link (visual search task, *r*_*s*_ = 0.48, p = 0.07; uncorrected; all other correlations: p > 0.14, uncorrected). We are, however, reluctant to make strong conclusions from this null result. Our study is underpowered for correlational analyses, especially when considering our heterogeneous group of participants.

### Learning retention 12 months after training

**Figure 7:**
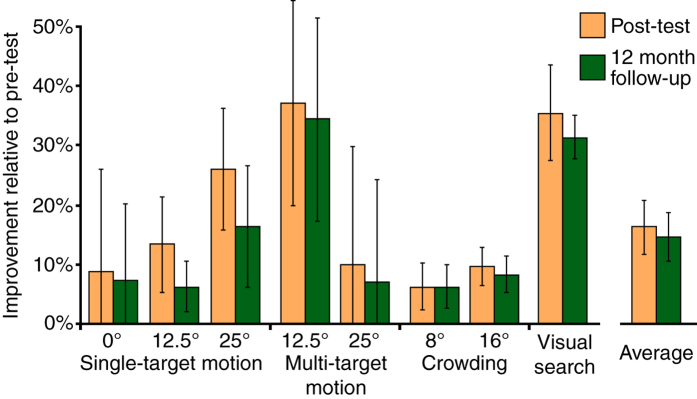
Learning retention 12 months after training. For four participants, we re-measured the key post-training measures at least 12 months after the end of training (2 for MAT and 2 for AVG). Data are expressed as % improvement relative to the pre-training baseline. The average across all tasks is shown on the far right. Error bars are SEM.

To examine whether training-induced improvements remained stable over time, we re-tested four LV participants 12 months after training (two from AVG group and two from MAT group). Given the small re-test sample and a lack of systematic group differences between AVG and MAT groups at post-training, we analysed AVG and MAT participants together. Results (Fig. 7) revealed that training-induced improvements remained relatively stable 12 months after training. Although, across tasks, we found a modest, but statistically reliable decline in task improvements (t_7_ = 3.19, p = 0.015), 12 month follow-up performance still exhibited highly significant improvements relative to the pre-training data (t_7_ = 4.1, p = 0.005).

## Discussion

Our results demonstrate that visual skills of LV youth can be enhanced by cognitive/perceptual training. Peripheral motion sensitivity, perceptual crowding, and visual search all improved after about eight hours of training. Notably, the training effects tended to be greater in the far periphery, with no improvements in the fovea. Several of these training effects were substantial. Importantly, preliminary evidence shows that the training effects appear to be generally stable after 12 months. A long-standing question in literature on action video games and plasticity is what game components primarily contribute to training-induced enhancements in perception and cognition^38^. Here, we showed that action video games were not necessary for achieving generalized perceptual learning. In fact, MAT training produced comparable (and often numerically larger) improvements as AVG training in tasks where improvements were observed.

What training aspects were instrumental in achieving the observed gains in perception? While our study was not designed to evaluate subcomponents of each training task, both AVG and MAT training likely developed skills in coordinating dynamic visual attention. MAT included two concurrent, dynamic, attention-demanding tasks: (A) Attending simultaneously to several independently moving objects required rapid switching and dividing attention to multiple objects at continually changing locations^36^,^39^; (B) A peripheral motion-discrimination task demanded concurrent vigilance for detecting and discriminating brief, spatially and temporally unpredictable, peripheral motion stimuli. Similar task demands also occur in action video games. However, the complexity of action video games makes it difficult to pinpoint the critical components responsible for observed training effects. Action video games place significant demands on the visual attention system, but also entail additional visual-motor demands^1^. As training in action video games and multiple object tracking have already been shown to effectively enhance spatial-temporal attention coordination^18^,^19^,^40^, we speculate this attentional enhancement, at least in part, accounts for improved performance noted in our visual tasks. This is also consistent with coupling effects of attention and learning in visual processing. Efficient attentional orienting can improve low-level stimulus detection and decrease response times, even when the eyes are looking elsewhere^41^. Previous experiments also showed that top-down attention plays an important role in supervising low-level perceptual learning^42^,^43^. Moreover, improvements in attention can also reduce the crowding effect^18^,^44^. Finally, as an important component of central executive function, improvements in attention can also affect high-level cognition, such as memory encoding and retrieval and executive control. In turn, these key aspects of high-level cognition may also affect learning. In fact, this is suggested by the “learn to learn” hypothesis, which explains broad transfer of action video game training as caused, at least in part, by enhancements in high-level learning ability^1^,^22^.

During natural vision, attention guides visual processing toward task-relevant and salient items in complex visual scenes. Better attention has been shown to substantially increase saliency detection, feature selection and integration^45^,^46^. Such improvements will likely have effects on everyday functioning for LV individuals, who commonly report difficulties in everyday tasks that rely on visual search skills, expressing problems with distracting objects and events, cluttered visual scenes, and inefficiencies in locating task relevant objects. Liu, Kuyk and Fuhr^6^ trained LV and TS subjects on a feature search task. Both groups improved after 5 days of training. Of interest, LV subjects improved more on a 40° field size than on 10° and 20° field sizes, while TS subjects did not show a difference by field size. This is consistent with the results of our study, namely evidence for larger improvements in far periphery.

The pattern of vision improvements reported here is of possible relevance for improving the effective visual span and reading speed. While reading is largely dependent on high-acuity central vision, it is also affected by peripheral function. Specifically, tolerance for peripheral perceptual crowding has important implications for reading and is a common practical problem for visually impaired individuals, including amblyopia or normal aging^47^,^48^,^49^,^50^. One review of studies reported that the primary presenting complaint at low vision clinics is problems with reading^51^. It appears that low-vision readers primarily differ from normal readers by having abnormally short saccades^52^. Notably, these abnormally short saccades can be linked with a reduction in the visual span of letters that can be recognized in one fixation^53^,^54^. Attention, in general, has also been linked with reading. A recent longitudinal study found that visual spatial attention in pre-schoolers predicts future reading acquisition^55^. Thus, it can be argued that reducing perceptual crowding and/or improving spatial resolution of attention may help to improve reading ability. Supporting this assertion, Franceschini, *et al*.^56^ found that action video game training can improve reading skill in dyslexic children. On the other hand, a visual crowding training study with typical individuals failed to find significant improvements in reading speed^57^. It is possible, however, that utilization of broader and more complex training regimens (e.g., action video game) may be more effective than use of very specific training paradigms (e.g., visual crowding training).

In sum, we studied LV patients who exhibited severe visual perception deficits that were especially prominent in the far periphery^32^. Results revealed that both AVG and MAT training were effective at reducing the observed deficits, especially in the far visual periphery. Given that improvements also included motion perception tasks, we speculate that the possible practical benefits also include improved visually guided mobility. We discuss these results in context of improved attentional functioning. These findings shed light on mechanisms that underline visual impairments in peripheral vision of LV individuals and suggest possible therapeutic interventions for their correction.

## Methods

### Participants

Twenty-four children and adolescents with low vision (ages 9–18; mean = 14.2 years) were recruited and tested at the Tennessee School for the Blind and the Oklahoma School for the Blind. Participants’ best-corrected binocular acuities ranged between 20/60 and 20/800. Participants were selected from a larger cohort (~300 students). Inclusion criteria included visual fields of at least 35° in both visual hemifields and no history of cognitive impairments. Detailed information about participants and their etiologies is in our previous publication^32^. All 24 participants completed the entire study. See Table 1 for clinical diagnoses, acuity, ages and group assignment of participants with low vision. A control group of 7 adolescents with typical visual fields and normal or corrected-to-normal acuity (ages 10–17; mean = 14.6 years) was tested at Vanderbilt University. The study was conducted in accordance with the tenets of the Declaration of Helsinki and was approved by Institutional Review Board at Vanderbilt University. All participants provided written informed consent.

Previous pilot work suggested that participant’s prior experience with visually demanding activities (e.g., action video games, certain ball-based sports, biking) could have a confounding effect on study measures. To control the influence of this factor on outcome measures, a randomized block design was incorporated into the assignment process. Participants were first assessed on previous relevant experiences with a questionnaire. Based on their answers, participants were stratified, or blocked, into three levels of this factor. Participants in each block level were then randomly assigned to a training condition without replacement. This process continued until all participants within a block had been assigned. This form of assignment ensures that each experience level is equally represented in each training condition.

### Apparatus

#### Pre/post training tasks and MAT training

All dependent measurements and MAT training tasks were created using the Psychophysics Toolbox^58^,^59^ and MATLAB (The MathWorks Inc., Natick, MA). All stimuli were shown at high contrast (99%). For the central acuity task only, stimuli were displayed on a linearized 19-inch liquid crystal display monitor (ViewSonic VX924; 1024 by 768 resolution, 85 Hz). Viewing was binocular at a distance of 77 cm, with individual pixels subtending 1.64 arcmin^2^. Ambient and background illumination levels were 0.13 and 33.5 cd/m^2^, respectively.

Stimuli used in all other pre/post tasks and for all training tasks were projected onto a matte screen (174 cm by 130 cm; 58° by 45°) by a linearized projector (NEC WT610; 1024 by 768 resolution, 120 Hz). Viewing distance was 156 cm, with individual pixels subtending 3.75 arcmin^2^. Ambient and background illumination levels were 0.04 and 46.3 cd/m^2^, respectively. In order to fit training sessions into participants’ school schedules, on several occasions we had to run two concurrent training sessions. For such cases, we used an additional projector set-up: linearized Panasonic AE-9000U projector (198 cm horizontal ×149 cm vertical area, with resolution of 1024 by 768, 85 Hz). Display parameters were made as similar as possible to the NEC projector. The viewing distance was ~177 cm, which resulted in similar pixel size (3.755 arcmin^2^) and viewing area (56° by 42°). Ambient and background illumination levels were 0.7 and 58 cd/m^2^, respectively. PlayStation 2 video game console was used for AVG and control training (more details below).

### Procedure

All pre/post tasks were computer-based and, with the exception of visual search, incorporated adaptive QUEST staircases^60^. Thresholds (82% correct) were estimated with blocks of 25 trials per threshold estimate. Pilot work showed that, when faced with a novel psychophysical task, our cohort of participants took several blocks of trials to master task demands. To get pre-training measures that are minimally affected by this initial learning stage, participants practiced each task until sequential thresholds did not vary by more than 15% (usually requiring 3–4 blocks of trials). Following practice, threshold estimates were obtained from three to five 25-trial blocks of trials. Auditory feedback signalled correct responses. Individual trials were self-paced, with the observer initiating the stimulus sequence by a key press.

Pre-training measures were given in the same order for all observers: (A) multi-target motion comparison, (B) crowding, (C) visual search, (D) single-target motion discrimination, and (E) central acuity for motion. This fixed task sequence was designed to reduce the effects of changing stimulus-response mappings between tasks, which was observed in our pilot work. For instance, when the motion comparison task followed motion direction discriminations, we found that observers would sometimes erroneously respond based on motion direction. Thus, we started with the comparison task, and placed search and crowding tasks between the two motion tasks to provide an additional “buffer.” With the exception of the visual search task, a large fixation cross was used before each trial (2.5° width/height). Eye movements were not recorded. Fixation compliance was informally verified by the experimenter throughout the experiment. For tasks that included peripheral stimuli (tasks A, B, and D), multiple randomized stimulus locations were used, evenly distributed between the left and the right visual field. This discourages anticipatory eye movements.

After completing pre-training task, participants were assigned to a training condition. For all three training groups, training consisted of playing their predetermined task for 10 total sessions (40–50 minutes per session, minimum of 3 times a week and maximum of 5 times a week). After the training phase was complete, participants repeated the same computer-based measurement tasks described above, recorded as post-training measures.

### Pre/post training measures

#### Central acuity for motion

This task effectively measures the high spatial frequency (SF) cut-off for motion direction discriminations of 10 Hz drifting gratings (i.e., the highest SF at which motion discrimination was possible). A participant’s task was to identify perceived motion direction (up or down) of centrally presented stimuli. Stimuli were presented for 150 ms (square-wave envelope) and shown in a spatial raised cosine window, whose size was adjusted to contain 2.25 grating cycles. That is, stimuli with higher SF were shown in smaller spatial envelopes.

#### Single-target motion direction discrimination

In this task, participants also identified motion direction (up or down) of briefly presented stimuli. However, the stimuli were presented in randomly varied locations. Thus, this task is similar to the frequently used *Useful Field of View* test^61^. Stimuli were Gabors: drifting gratings shown in stationary Gaussian spatial envelopes (2σ = 3.2°). For this and all tasks described below, a fixed stimulus size was used (i.e., stimuli were not scaled for eccentricity). Stimulus motion was either upward or downward (13.3°/sec, 10 Hz). SF was 0.75 cycles/°, low enough to ensure visibility over the full range of eccentricities used in this experiment. To measure visual sensitivity, we used QUEST staircases to estimate the briefest presentation durations sufficient for accurate perception of stimulus motion direction^62^,^63^. Stimulus duration was set by a hybrid Gaussian envelope^64^, with duration taken as the width at half height of the temporal envelope. Stimulus location was unpredictable. There were 13 possible stimulus locations, with stimulus presentations distributed evenly between three eccentricities (0°, ±12°, ±25°) and three radial axes (horizontal, 45°/225°, 135°/315°). Thresholds were estimated for each of three eccentricities, collapsing across radial axes.

#### Multi-target motion direction comparison

This task requires participants to perceptually compare motions of three spatially separate stimuli, presented simultaneously in the left, central and right visual fields. Stimulus motions could be either all identical (i.e., either all upward or all downward), or, on a half of the trials, one stimulus direction was opposite from the other two (e.g., two downward and one upward). Participants discriminated between these two types of trials (all motions same vs. one oddball). There were two randomly interleaved spatial arrangements. While the middle stimulus was always presented in the centre, the two peripheral stimuli were either at ±12° or ±25° eccentricity along the horizontal meridian. All other details are as described for the single-target experiment.

#### Visual crowding

Crowding occurs when a group of objects is shown in visual periphery, resulting in impaired discrimination of the “crowded” object^37^. This effect becomes more pronounced as the eccentricity increases. Consequently, crowding effectively limits visual spatial resolution in cluttered scenes, limiting critical visual functions such as reading and object recognition. We measured crowding with stimuli composed of five neighbouring Landolt C-shapes (3° diameter, with a 45° wide opening). Target shape was in the centre, surrounded by four equidistant distracter shapes. The participant’s task was to identify the direction of the Landolt C opening (up, down, left, right). This stimulus briefly appeared as one of four randomly selected locations (±8° or ±16° eccentricity, randomly selected). To measure thresholds, we used QUEST staircases to adjust the centre-to-centre separation between target and distracters. Larger spacing thresholds indicate worse performance and greater susceptibility to crowding.

#### Naturalistic Visual Search

Participants were asked to locate everyday objects (e.g., coffee cup, plant, telephone) within photographs of everyday scenes (e.g., office rooms). We estimated visual search ability using images of target objects in cluttered scenes (e.g., a stapler in a cluttered office). Thirty-six unique target/scene-combinations were created with nine target objects (e.g., stapler, medicine bottle), each presented in four different scenes (e.g., work office). Stimuli were shown on the large projection screen (as described above) allowing for objects and scenes to be close to life-size. Target object size ranged from 3° to 9° (median ~5°). Each target-scene pair was presented once in a set of 36 trials. Different sets of images were used for pre- and post-training.

On each trial, the target object was first shown in isolation (5 s) and was verbally identified by the experimenter. Next, the visual search scene appeared. Participants were instructed to first visually locate the target object (i.e., perform visual search), and then use a laser pointer to mark its location. The laser pointer action was accurate, fast and direct. That is, the reported search times reflect variability in visual search times and not individual difficulties in the pointing action. If the target was correctly localized, the experimenter pushed a button to stop the trial and the timer. In case of the incorrect localizations, the experimenter only said “no,” which indicated to the participant to keep searching. If 30 s elapsed, the experimenter said “Keep looking, you’ll find it.” If the target was not located in 30 sec, the experimenter restated the name of the target.

### Training Tasks

For AVG training, we used a PlayStation 2 game called “Ratchet and Clank: Dreadlocked”. This game includes the task demands characteristic of action video games used in previous studies but it is more appropriate for children and adolescent participants than typically used first-person shooter games. The control task is a video game called “Lumines.” This game is similar to the well-known game “Tetris,” which has been used regularly in previous training studies as a control condition^65^.

The MAT training task is a modified multiple-object tracking task. Participants tracked moving targets embedded in a field of competing, and visually identical, distracting elements. During target tracking, temporally and spatially unpredictable Gabors are presented in the far periphery. Participants are asked to discriminate the motion (up/down) of these Gabors.

**Figure 8:**
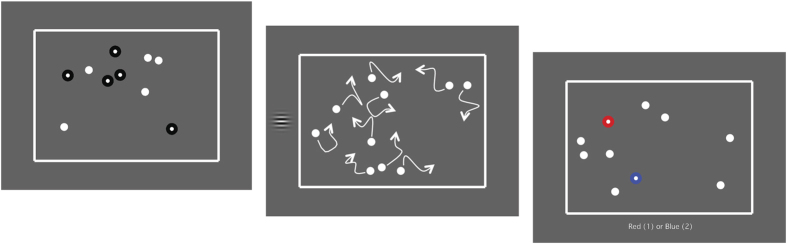
An illustration of a typical trial during MAT training. First, target balls to be tracked are briefly identified with a black outline (left panel). The black outlines disappear, and all balls start to move in random directions (centre panel). The participant’s task is to track the target balls. During the tracking task, drifting Gabor targets appear on either the right or the left side. The participant’s task is to also discriminate Gabor motion direction. After 10 seconds, all balls stop moving and two are highlighted (right panel). The participant’s task is to identify which ball was among the target balls.

In a typical trial (Fig. 8), participants hit a key to initiate the trial sequence. A number of static white balls (4–10; 1.5° in diameter) are presented for 2 seconds, appearing in random locations within a box (46° wide × 34° high). During this time, 2–5 balls are highlighted with a black outline to signal their status as targets. Next, all balls become visually identical and begin moving randomly and independently of one another, avoiding each other and the edge of the box. These balls can approach but never touch each other (they repulse each other). Movement continues for 10 seconds, during which time briefly presented Gabors may appear directly outside the box to the left or right (+/− 25° eccentricity). Participants are asked to discriminate their motion (up or down; all other task details as described for the Single-target motion direction discrimination task). Between 0 and 2 Gabor presentations may occur during a single tracking trial. Onset of the first Gabor (if present) occurs sometimes between 0.5 and 3.5 s after the beginning of target tracking. Onset of the second Gabor (if present) occurs between 0.5 and 3.5 s after the response to the first Gabor. After balls stop moving, two balls become highlighted (one red and another blue; Fig. 8, far right panel). Participants then respond to which highlighted ball is a target. Immediate auditory feedback is provided for correct or incorrect answers to both the tracking task and the motion discrimination task.

Two QUEST staircase procedures are used in the MAT training task. One adjusts the velocity of ball targets and distractors. Velocities typically ranged between 5 and 30°/s. If ball velocity exceeded 25°/s, then the number of targets and distractors was increased for the next block of trials. Participants usually completed two blocks of 50 trials during each training session. A second QUEST procedure adjusted durations of the peripheral targets (as described for the Single-target motion direction discrimination task). These two QUEST procedures were used to keep performance adapted to participants’ skill level, similar to video games. By converging toward 82% correct threshold, this minimizes the experience of failure, which presumably should have a positive effect on participants’ motivation.

### Data Analysis

As expected given diverse etiologies and varied acuity among participants (Table 1), we observed very high individual variability across all of the pre-training measures^32^. Such baseline differences make it very challenging to directly evaluate the training effects under different regimens. Thus, we utilized analysis of covariance (ANCOVA/MANCOVA) in which extra covariates, presumably the source of variability, are included. Analysis of covariance allowed us to directly disentangle training effects from various sources of theoretically unrelated factors and to address how different groups of subjects improved when the pre-training baseline was statistically controlled. All pairwise comparisons are Bonferroni corrected.

## Additional Information

**How to cite this article**: Nyquist, J. B. *et al*. Perceptual training yields rapid improvements in visually impaired youth. *Sci. Rep.* **6**, 37431; doi: 10.1038/srep37431 (2016).

**Publisher's note:** Springer Nature remains neutral with regard to jurisdictional claims in published maps and institutional affiliations.

## Figures and Tables

**Table 1:** Ages and Visual Characteristics of Participants.

Age	Binocular acuity	Clinical Diagnosis	Nystagmus
Control group			
14	20/400	Nystagmus	Yes
14	20/200	Ocular albinism	Yes
14	20/200	Ocular albinism	Yes
10	20/800	Ocular albinism	Yes
13	20/200	Ocular albinism, photophobia	Yes
9	20/200	Retinopathy of prematurity	No
15	20/400	Retinopathy of prematurity	No
10	20/200	Stargardt’s macular dystrophy	No
14	20/60	Stargardt’s macular dystrophy	No
Action video game group			
14	20/200	Aphakia, chronic blepharitis, juvenile glaucoma	No
15	20/200	Bilateral iris, retinal colobomas	No
16	20/200	Congenital cataracts, aphakia	Yes
10	20/300	Esotropia	Yes
16	20/400	Retinitis pigmentosa	No
13	20/200	Septo-optic dysplasia	No
18	20/200	Stargardt’s macular dystrophy	No
15	20/200	Stargardt’s macular dystrophy	No
Modified attentional tracking group			
15	20/200	Congenital cataracts, aphakia	Yes
15	20/200	Hyperopia	Yes
17	20/400	Ocular albinism	Yes
16	20/800	Ocular albinism	Yes
16	20/400	Ocular albinism, exotropia	Yes
16	20/200	Ocular albinism, photophobia	Yes
15	20/800	Retinopathy of prematurity	No

## References

[b1] Bavelier D., Green C. S., Pouget A. & Schrater P. Brain plasticity through the life span: learning to learn and action video games. Annu. Rev. Neurosci. 35, 391–416 (2012). 22715883 10.1146/annurev-neuro-060909-152832

[b2] Watanabe T. & Sasaki Y. Perceptual learning: toward a comprehensive theory. Annu. Rev. Psychol. 66, 197–221 (2015). 25251494 10.1146/annurev-psych-010814-015214 PMC4286445

[b3] Blackwell D. L., Lucas J. W. & Clarke T. C. Summary health statistics for U.S. adults: national health interview survey, 2012. Vital Health Stat. 10, 1–161 (2014). 24819891

[b4] Bishop V., Corn A. & Koenig A. Foundations Of Low Vision: Clinical And Functional Perspectives. (AFB Press New York, 1996).

[b5] Li R. W., Young K. G., Hoenig P. & Levi D. M. Perceptual learning improves visual performance in juvenile amblyopia. Invest. Ophthalmol. Vis. Sci. 46, 3161–3168 (2005). 16123415 10.1167/iovs.05-0286

[b6] Liu L., Kuyk T. & Fuhr P. Visual search training in subjects with severe to profound low vision. Vision Res. 47, 2627–2636 (2007). 17707452 10.1016/j.visres.2007.07.001

[b7] Polat U., Ma-Naim T., Belkin M. & Sagi D. Improving vision in adult amblyopia by perceptual learning. Proc Natl Acad Sci USA 101, 6692–6697 (2004). 15096608 10.1073/pnas.0401200101 PMC404107

[b8] Karni A. & Sagi D. Where practice makes perfect in texture discrimination: evidence for primary visual cortex plasticity. Proc Natl Acad Sci USA. 88, 4966–4970 (1991). 2052578 10.1073/pnas.88.11.4966 PMC51788

[b9] Xiao L. Q. . Complete transfer of perceptual learning across retinal locations enabled by double training. Curr. Biol. 18, 1922–1926 (2008). 19062277 10.1016/j.cub.2008.10.030 PMC3045109

[b10] Lev M. . Training improves visual processing speed and generalizes to untrained functions. Sci. Rep. 4, 7251 (2014). 25431233 10.1038/srep07251 PMC4246693

[b11] Huang C. B., Zhou Y. & Lu Z. L. Broad bandwidth of perceptual learning in the visual system of adults with anisometropic amblyopia. Proc Natl Acad Sci USA. 105, 4068–4073 (2008). 18316716 10.1073/pnas.0800824105 PMC2268837

[b12] Das A., Tadin D. & Huxlin K. R. Beyond blindsight: properties of visual relearning in cortically blind fields. J. Neurosci. 34, 11652–11664 (2014). 25164661 10.1523/JNEUROSCI.1076-14.2014 PMC4145170

[b13] Melnick M. D., Tadin D. & Huxlin K. R. Relearning to See in Cortical Blindness. Neuroscientist. 22, 199–212 (2016). 26659828 10.1177/1073858415621035 PMC4795973

[b14] Astle A. T., Li R. W., Webb B. S., Levi D. M. & McGraw P. V. A Weber-like law for perceptual learning. Sci. Rep. 3, 1158 (2013). 23362458 10.1038/srep01158 PMC3557449

[b15] Polat U. Restoration of underdeveloped cortical functions: evidence from treatment of adult amblyopia. Restor. Neurol. Neurosci. 26, 413–424 (2008). 18997316

[b16] Yehezkel O., Sterkin A., Lev M., Levi D. M. & Polat U. Gains following perceptual learning are closely linked to the initial visual acuity. Sci. Rep. 6, 25188 (2016). 27122254 10.1038/srep25188 PMC4848560

[b17] Dye M. W. & Bavelier D. Playing video games enhances visual attention in children. Journal of Vision. 4, 40–40 (2004).

[b18] Green C. S. & Bavelier D. Effect of action video games on the spatial distribution of visuospatial attention. J. Exp. Psychol. Hum. Percept. Perform. 32, 1465–1478 (2006). 17154785 10.1037/0096-1523.32.6.1465 PMC2896828

[b19] Green C. S. & Bavelier D. Action video game modifies visual selective attention. Nature. 423, 534–537 (2003). 12774121 10.1038/nature01647

[b20] Castel A. D., Pratt J. & Drummond E. The effects of action video game experience on the time course of inhibition of return and the efficiency of visual search. Acta Psychol. (Amst). 119, 217–230 (2005). 15877981 10.1016/j.actpsy.2005.02.004

[b21] Li R., Polat U., Makous W. & Bavelier D. Enhancing the contrast sensitivity function through action video game training. Nat. Neurosci. 12, 549–551 (2009). 19330003 10.1038/nn.2296 PMC2921999

[b22] Bejjanki V. R. . Action video game play facilitates the development of better perceptual templates. Proc Natl Acad Sci USA. 111, 16961–16966 (2014). 25385590 10.1073/pnas.1417056111 PMC4250112

[b23] Vedamurthy I. . Recovering stereo vision by squashing virtual bugs in a virtual reality environment. Philos. Trans. R. Soc. Lond. B Biol. Sci. 371 (2016). 10.1098/rstb.2015.0264 PMC4901458 27269607

[b24] Vedamurthy I., Nahum M., Bavelier D. & Levi D. M. Mechanisms of recovery of visual function in adult amblyopia through a tailored action video game. Sci. Rep. 5, 8482 (2015). 25719537 10.1038/srep08482 PMC4894407

[b25] Vedamurthy I. . A dichoptic custom-made action video game as a treatment for adult amblyopia. Vision Res. 114, 173–187 (2015). 25917239 10.1016/j.visres.2015.04.008 PMC4549206

[b26] To L. . A game platform for treatment of amblyopia. IEEE Trans. Neural Syst. Rehabil. Eng. 19, 280–289 (2011). 21335317 10.1109/TNSRE.2011.2115255

[b27] Li R. W., Ngo C., Nguyen J. & Levi D. M. Video-game play induces plasticity in the visual system of adults with amblyopia. PLoS Biol. 9, e1001135 (2011). 21912514 10.1371/journal.pbio.1001135 PMC3166159

[b28] Subrahmanyam K. & Greenfield P. M. Effect of video game practice on spatial skills in girls and boys. J. Appl. Dev. Psychol. 15, 13–32 (1994).

[b29] Sims V. K. & Mayer R. E. Domain specificity of spatial expertise: the case of video game players. Appl. Cogn. Psychol. 16, 97–115 (2002).

[b30] Ambrose G. V. & Corn A. L. Impact of low vision on orientation: an exploratory study. RE: view. 29, 80 (1997).

[b31] Ludt R. & Goodrich G. L. Change in visual perceptual detection distances for low vision travelers as a result of dynamic visual assessment and training. Journal of Visual Impairment & Blindness. 96, 7–21 (2002).

[b32] Tadin D., Nyquist J. B., Lusk K. E., Corn A. L. & Lappin J. S. Peripheral vision of youths with low vision: motion perception, crowding, and visual search. Invest. Ophthalmol. Vis. Sci. 53, 5860–5868 (2012). 22836766 10.1167/iovs.12-10350 PMC3428114

[b33] Peli E. Vision multiplexing: an engineering approach to vision rehabilitation device development. Optom. Vis. Sci. 78, 304–315 (2001). 11384008 10.1097/00006324-200105000-00014

[b34] Weldon K. B., Rich A. N., Woolgar A. & Williams M. A. Disruption of Foveal Space Impairs Discrimination of Peripheral Objects. Front. Psychol. 7, 699 (2016). 27242612 10.3389/fpsyg.2016.00699 PMC4862972

[b35] Pylyshyn Z. W. & Storm R. W. Tracking multiple independent targets: evidence for a parallel tracking mechanism. Spat. Vis. 3, 179–197 (1988). 3153671 10.1163/156856888x00122

[b36] Scholl B. J., Pylyshyn Z. W. & Feldman J. What is a visual object? evidence from target merging in multiple object tracking. Cognition. 80, 159–177 (2001). 11245843 10.1016/s0010-0277(00)00157-8

[b37] Levi D. M. Crowding–an essential bottleneck for object recognition: a mini-review. Vision Res. 48, 635–654 (2008). 18226828 10.1016/j.visres.2007.12.009 PMC2268888

[b38] Green C. S. & Bavelier D. Learning, attentional control, and action video games. Curr. Biol. 22, R197–R206 (2012). 22440805 10.1016/j.cub.2012.02.012 PMC3461277

[b39] Sears C. R. & Pylyshyn Z. W. Multiple object tracking and attentional processing. Can. J. Exp. Psychol. 54, 1–14 (2000). 10721235 10.1037/h0087326

[b40] Makovski T., Vazquez G. A. & Jiang Y. V. Visual learning in multiple-object tracking. PLoS One. 3, e2228 (2008). 18493599 10.1371/journal.pone.0002228 PMC2375111

[b41] Carrasco M. Visual attention: the past 25 years. Vision Res. 51, 1484–1525 (2011). 21549742 10.1016/j.visres.2011.04.012 PMC3390154

[b42] Ito M., Westheimer G. & Gilbert C. D. Attention and perceptual learning modulate contextual influences on visual perception. Neuron. 20, 1191–1197 (1998). 9655506 10.1016/s0896-6273(00)80499-7

[b43] Li W., Piech V. & Gilbert C. D. Perceptual learning and top-down influences in primary visual cortex. Nat. Neurosci. 7, 651–657 (2004). 15156149 10.1038/nn1255 PMC1440483

[b44] Poder E. Effect of attention on the detection and identification of masked spatial patterns. Perception. 34, 305–318 (2005). 15895629 10.1068/p5276

[b45] Wolfe J. M. Guided Search 2.0 A revised model of visual search. Psychon Bull Rev. 1, 202–238 (1994). 24203471 10.3758/BF03200774

[b46] Peelen M. V. & Kastner S. Attention in the real world: toward understanding its neural basis. Trends Cogn. Sci. 18, 242–250 (2014). 24630872 10.1016/j.tics.2014.02.004 PMC4908952

[b47] Ball K. K., Beard B. L., Roenker D. L., Miller R. L. & Griggs D. S. Age and visual search: expanding the useful field of view. J. Opt. Soc. Am. A. 5, 2210–2219 (1988). 3230491 10.1364/josaa.5.002210

[b48] Martelli M., Di Filippo G., Spinelli D. & Zoccolotti P. Crowding, reading, and developmental dyslexia. J Vis. 9, 14 11–18 (2009). 10.1167/9.4.14 19757923

[b49] Yu D., Cheung S. H., Legge G. E. & Chung S. T. Effect of letter spacing on visual span and reading speed. J Vis. 7, 2 1–10 (2007). 10.1167/7.2.2 PMC2729067 18217817

[b50] Pelli D. G. . Crowding and eccentricity determine reading rate. J Vis. 7, 20 21–36 (2007). 18217835 10.1167/7.2.20

[b51] Leat S. J., Legge G. E. & Bullimore M. A. What is low vision? A re-evaluation of definitions. Optom. Vis. Sci. 76, 198–211 (1999). 10333182 10.1097/00006324-199904000-00023

[b52] Rumney N., Leat S. & Ave K. E. V. Why do low vision patients still read slowly. Low vision: Research and new developments in rehabilitation. 11, 269 (1994).

[b53] Legge G. E., Ahn S. J., Klitz T. S. & Luebker A. Psychophysics of reading—XVI. The visual span in normal and low vision. Vision Res. 37, 1999–2010 (1997). 9274784 10.1016/s0042-6989(97)00017-5

[b54] Legge G. E., Mansfield J. S. & Chung S. T. Psychophysics of reading. XX. Linking letter recognition to reading speed in central and peripheral vision. Vision Res. 41, 725–743 (2001). 11248262 10.1016/s0042-6989(00)00295-9

[b55] Franceschini S., Gori S., Ruffino M., Pedrolli K. & Facoetti A. A causal link between visual spatial attention and reading acquisition. Curr. Biol. 22, 814–819 (2012). 22483940 10.1016/j.cub.2012.03.013

[b56] Franceschini S. . Action video games make dyslexic children read better. Curr. Biol. 23, 462–466 (2013). 23453956 10.1016/j.cub.2013.01.044

[b57] Chung S. T. Learning to identify crowded letters: does it improve reading speed? Vision Res. 47, 3150–3159 (2007). 17928026 10.1016/j.visres.2007.08.017 PMC2134936

[b58] Brainard D. H. The Psychophysics Toolbox. Spat. Vis. 10, 433–436 (1997). 9176952

[b59] Pelli D. G. The VideoToolbox software for visual psychophysics: transforming numbers into movies. Spat. Vis. 10, 437–442 (1997). 9176953

[b60] Watson A. B. & Pelli D. G. QUEST: a Bayesian adaptive psychometric method. Percept. Psychophys. 33, 113–120 (1983). 6844102 10.3758/bf03202828

[b61] Ball K., Owsley C. & Beard B. Clinical visual perimetry underestimates peripheral field problems in older adults. Clinical Vision Sciences. 5, 113–125 (1990).

[b62] Lappin J. S., Tadin D., Nyquist J. B. & Corn A. L. Spatial and temporal limits of motion perception across variations in speed, eccentricity, and low vision. J Vis. 9, 30 31–14 (2009). 10.1167/9.1.30 19271900

[b63] Tadin D., Lappin J. S., Gilroy L. A. & Blake R. Perceptual consequences of centre-surround antagonism in visual motion processing. Nature. 424, 312–315 (2003). 12867982 10.1038/nature01800

[b64] Tadin D., Silvanto J., Pascual-Leone A. & Battelli L. Improved motion perception and impaired spatial suppression following disruption of cortical area MT/V5. J. Neurosci. 31, 1279–1283 (2011). 21273412 10.1523/JNEUROSCI.4121-10.2011 PMC3078722

[b65] Amitay S., Irwin A. & Moore D. R. Discrimination learning induced by training with identical stimuli. Nat. Neurosci. 9, 1446–1448 (2006). 17028582 10.1038/nn1787

